# A Biological Immunity-Based Neuro Prototype for Few-Shot Anomaly Detection with Character Embedding

**DOI:** 10.34133/cbsystems.0086

**Published:** 2024-01-16

**Authors:** Zhongjing Ma, Zhan Chen, Xiaochen Zheng, Tianyu Wang, Yuyang You, Suli Zou, Yu Wang

**Affiliations:** ^1^ School of Automation, Beijing Institute of Technology, Beijing 100081, China.; ^2^ ETH AI Center, Andreasstrasse 5, 8092 Zürich, Switzerland.; ^3^ State Key Lab of Multimodal Artificial Intelligence Systems, Institute of Automation, Chinese Academy of Sciences, Beijing 100095, China.

## Abstract

Anomaly detection has wide applications to help people recognize false, intrusion, flaw, equipment failure, etc. In most practical scenarios, the amount of the annotated data and the trusted labels is low, resulting in poor performance of the detection. In this paper, we focus on the anomaly detection for the text type data and propose a detection network based on biological immunity for few-shot detection, by imitating the working mechanism of the immune system of biological organisms. This network enabling the protected system to distinguish the aggressive behavior of “nonself” from the legitimate behavior of “self” by embedding characters. First, it constructs episodic task sets and extracts data representations at the character level. Then, in the pretraining phase, Word2Vec is used to embed the representations. In the meta-learning phase, a dynamic prototype containing encoder, routing, and relation is designed to identify the data traffic. Compare to the mean-based prototype, the proposed prototype applies a dynamic routing algorithm that assigns different weights to samples in the support set through multiple iterations to obtain a prototype that combines the distribution of samples. The proposed method is validated on 2 real traffic datasets. The experimental results indicate that (a) the proposed anomaly detection prototype outperforms state-of-the-art few-shot techniques with 1.3% to 4.48% accuracy and 0.18% to 4.55% recall; (b) under the premise of ensuring the accuracy and recall, the number of training samples is reduced to 5 or 10; (c) ablation experiments are designed for each module, and the results show that more accurate prototypes can be obtained by using the dynamic routing algorithm.

## Introduction

Text data analysis can effectively help us understand the data corpus, quickly identify potential problems in the data, and guide subsequent model training and selection. This kind of data is widely presented in networks, Internet, logs, devices, and operating systems. In order to find the anomalies in the data and prevent the damage to the system, an anomaly detection (AD) has been used as one of the most critical systems [1]. For example, log AD refers to finding abnormal logs to determine the cause and nature of system faults. Usually, log data is modeled as a natural language sequence for AD.

Machine learning and deep learning are widely leveraged in the field of AD, hoping to improve the performance of AD systems, such as misuse based detection [2], deception based detection, and bio-based detection [3]. Machine learning uses algorithms to parse network data, learn characteristics of traffic data, and then classify and predict a certain class of things. Classic machine learning models, such as random forest [4], support vector machine (SVM) [5], and Adaboost [6], have been introduced to detect anomalies. Horng et al. [7] proposed an AD system based on SVM, in which a hierarchical clustering algorithm was used to deal with typical data. It reduced the complexity and redundancy of the dataset and further reduced the training time. Al-Yaseen et al. [8] proposed a multilevel hybrid detection model based on SVM, with the results showing an accuracy of 95.75%. In [9], a network AD system was presented based on Light GBM. They use an oversampling technique to increase minority samples of imbalanced training data to increase the detection accuracy. Since the detection performance of machine-learning-based algorithms is related to the manual selection of features, it requires plenty of professional knowledge to deeply mine the deep features in the data.

The deep-learning-based algorithms are to learn the inherent distribution and representation level of sample data and can automatically extract the characteristics of data such as text, images and sounds. At the same time, the nonlinear hidden layer structure in the neural network is helpful for the learning and prediction of high-dimensional data. In this context, deep learning algorithms are gradually considered for detection tasks, such as autoencoders [10,11], convolutional neural networks (CNNs) [12,13] and long short-term memory networks (LSTMs) [14]. For example, Min et al. [2] proposed a system that combined Text-CNN and random forest to construct an anomaly-based network detection system. Kim et al. [15] used deep learning to generate virtual samples on the dataset, and proposed a malware detection method based on deep convolutional generative adversarial networks. The role of generative adversarial network is to generate similar data to detect the deformation of malware more accurately. In [16], it applied a deep belief neural network to extract data features, and then use the backward propagation neural network as a classifier to identify traffic data anomalies. In [17], it proposed an AD method based on BiLSTM deep learning. Experiments showed that in binary and multiclass AD, BiLSTM not only improved the performance of traditional LSTM but also had higher detection accuracy.

An AD based on deep learning is essentially a classifier trained on a large amount of data, highly dependent on feature engineering and dataset capacity. Data imbalance and less training data often appear in reality, which will cause overfitting, falling into local optimal solutions, or other problems. Concerning about these problems, some researchers have tried to introduce few-shot learning prototype into network AD in recent years, such as [18–27]. Yu et al. [28] used the deep neural network (DNN) and CNN as the traffic embedding network to map each sample into a high-dimensional sample space. After that, the prototype vector of each anomaly category is obtained by averaging. Finally, the distance between the new anomaly and each prototype vector is measured to obtain the classification result. Rong et al. [25] proposed a few-shot learning based prototype UMVD-FSL to detect unseen malware variants with a small set of data. Start with network flow data generated by malware variants and benign applications, then convert them to grayscale images. A prototype-based few-shot learning model takes grayscale images as input and leverages meta-training to generalize the meta-learner to adapt to new tasks. Xu et al. [18] designed a deep neural network, which is mainly composed of 2 parts: feature extraction network and comparison network, to classify network traffic samples. Guo et al. [26] integrated a global attention mechanism and aggregated the global information of inputs by capturing the byte relationship between payload sequence. A metric-based AD prototype was proposed in [27], which makes feature extractor fuse original bytes content with network flow features to improve detection precision and recall. As noted by Sung et al., RelationNet [21] builds a learnable nonlinear comparator through neural network to calculate the distance between 2 samples and then analyzes the sample similarity, instead of a fixed linear comparator such as Euclidean distance or cosine distance. In general, the above methods apply mean-based prototypes to measure similarity by Euclidean distance to obtain classification results. However, the nearest-neighbor classifier based on mean prototype and the fixed linear comparator easily cause the estimation bias due to the data scarcity in few-shot scenarios and finally affect detection precision and recall. What is more, because of the hindrance of data parsing, building a universal network system to detect network traffic attacks is still a tough task for deep learning methods.

In this paper, we leverage the the working mechanism of the immune system to design a neural prototype for few-shot AD with character embedding, namely CharNet, which combines text embedding techniques and improved metric-based few-shot learning, improving the accuracy and recall of the existing deep models for few-shot network traffic classification. Specifically, CharNet consists of dataset construction, pretraining, meta-learning 3 phases. Dataset construction phase is to transform the original traffic dataset into a episode-based dataset, converting the multiclassification problem into a 2-way *K*-shot problem. To skip the data parsing session, Word2Vec, a self-supervised learning method, is used in the pretraining phase to convert traffic into embedding vectors. Therefore, the multidimensional feature data classification task is transformed into a text classification task. In the meta-learning phase, we design a dynamic routing prototype network, which consists of 3 modules: encoder, routing, and relation, to identify whether the network traffic is normal or not. Compare to the mean-based prototype, CharNet uses a dynamic routing algorithm that assigns different weights to samples in the support set through multiple iterations to obtain a prototype that combines the distribution of samples. At last, the relation module gives the classification results by properly performing comparison between those traffic embedding vectors. The main contributions of this paper are as follows:

• We propose the CharNet for few-shot AD. This method uses the dynamic routing algorithm to assign weights to the samples in the support set and builds a routing-based prototype, which effectively reduces the estimation bias and sampling bias caused by a small number of samples.

• We treat network traffic as a string and use character-level coding to omit data parsing sessions. This processing method can use the Word2Vec method to pretrain the embedding layer, which can learn prior knowledge for network traffic classification, effectively improving the training speed and accuracy.

• Compare our method with other methods, the accuracy of our method has improved. Using the proposed method, new types of samples on the basis of only a limited number of labels in an untrained dataset can be detected relying on learned prior knowledge.

The rest of the paper is organized as follows. Materials and Methods reviews the related work to our method. Experiments describes the prototype and details on our proposed prototype and presents our experiments and make comparisons with big-data methods and other few-shot learning methods. Conclusion summarizes the paper and make the conclusion.

## Materials and Methods

### Problem formulation

**Fig. 1.**
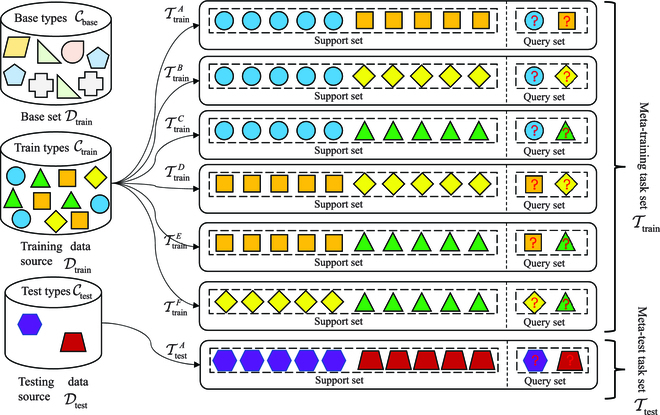
The illustration of the division of meta-learning tasks.

We consider network AD as the task of few-shot classifier learning, whose purpose is to train a classifier *f_θ_*(⋅) with few samples *x_i_* and predict the corresponding labels ${\hat{y}}_{i}$ of new samples ${\hat{x}}_{i}$. As shown in Fig. 1, we have 3 datasets: a base set $D_{\text{base}}$, a meta-training task set $T_{\text{train}}=\left\{S_{\text{train}},Q_{\text{train}}\right\}$_,_ and a meta-test task set $T_{\text{test}}=\left\{S_{\text{test}},Q_{\text{test}}\right\}$. Base set has a large number of samples with a set of classes $C_{\text{base}}$. If the support set of meta task sets contain *K*-labeled samples for each of *N* unique classes, the target few-shot problem is called *N*-way *K*-shot. As the project’s goal is usually to distinguish between normal samples and a particular type of malicious samples, we consider the network AD as a 2-way *K*-shot problem.

According episode-based training proposed in [29], in each episode iteration, the support set S is formed by *K*-labeled samples from each of the *C* classes, that is, $S={\left\{(x_{i},y_{i})\right\}}_{i=1}^{K\times C}$. Similarly, the query set Q is formed from the remainder of those *C* classes’ samples, that is, $Q={\left\{(x_{j},y_{j})\right\}}_{j=1}^{B}$. Meanwhile, the base set with abundant labeled samples $D_{\text{base}}={\left\{(x_{i},y_{i})\right\}}_{i=1}^{m}$. Both meta task sets contain the support set and the query set, and the support set and query set share the same label space, but the label space of meta-training task set is disjoint with the the label space of meta-test task set. That is, $C_{\text{train}}\cap C_{\text{test}}=Ø$, $S_{\text{train}}\cap Q_{\text{train}}=Ø$_,_ and $S_{\text{test}}\cap Q_{\text{test}}=Ø$.

Our goal is to learn a good meta-learner *f_θ_*(⋅) on the support set S based on prior knowledge obtained on the base set $D_{\text{base}}$ so that it can perform well on the query set Q. In our few-shot experiments (see Results and Discussion), we consider 5-shot (*K* = 5) and ten-shot (*K* = 10) settings.

### Overall prototype

**Fig. 2.**
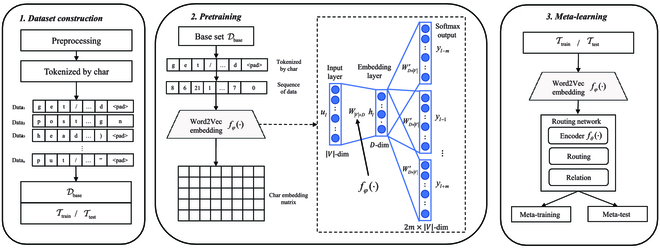
The prototype of few-shot AD.

As shown in Fig. 2, the proposed prototype is divided into 3 phases, including dataset construction, pretraining, and meta-learning.

#### Dataset construction

In the phase, we will construct base set $D_{\text{base}}$, train task set $T_{\text{train}}$_,_ and test task set $T_{\text{test}}$. We firstly perform preprocessing operations, such as duplicate value deletion, default value supplementation, and zero padding at the end of the most extended traffic log length. Then, each traffic log is tokenized by character level to extract fine-grained feature expressions. After that, in order to define each task as a 2-way *K*-shot task, we mix *K* normal samples with *K* malicious samples of each type.

The classes $C_{\text{train}}$ with a large number of samples are selected as the train task set $T_{\text{train}}$, and the remaining classes $C_{\text{test}}$ is used as the test task set $T_{\text{test}}$. To follow the episode-based strategy, the few-shot task generating details are shown in Algorithm 1. In order to improve the training speed and accuracy, we take the support set of train task set $T_{\text{train}}\langle S\rangle$ as the base set $D_{\text{base}}$ to participate in pretraining.

#### Pretraining

A Word2Vec [30] self-supervised classifier is trained with the samples in base set $D_{\text{base}}$. Skip-gram model is applied in our word-embedding task, which predicts the surrounding words from the central word. We define 2 parameter matrices, *W* ∈ ℝ^*D*×|*V*|^ and *W*^′^ ∈ ℝ^|*V*|×*D*^, where *D* represents the embedding dimension and can be set to any size. Since we treat network traffic as a string, we tokenize the string by characters [31] and one-hot encode it according to the dictionary *V* to get the sequence *u* = {*u*_1_, *u*_2_, …, *u_L_*}, *L* is the length of the sequence. Here, |*V*| is the size of the dictionary set *V*. Skip-gram works as the following steps: 
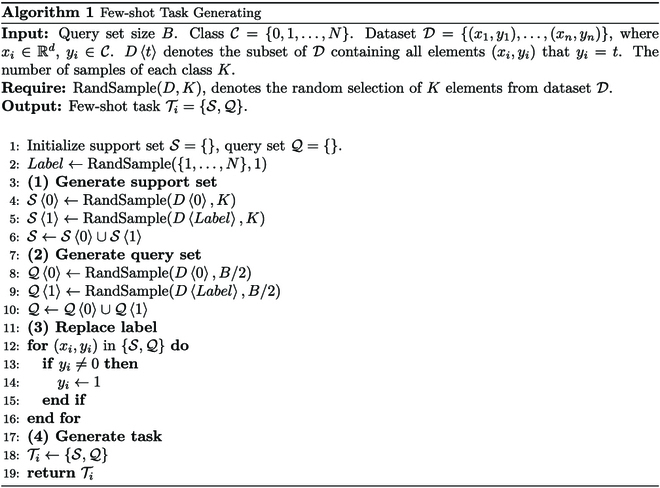


#### Meta-learning

In this phase, a meta-learner *f_θ_*(⋅) learn meta knowledge in $C_{\text{train}}$, and they can learn quickly and accurately with few data in $C_{\text{test}}$. This phase is divided in 2 phases: meta-training and meta-test. In Dataset construction, we have constructed a train task set $T_{\text{train}}$ and a test task set $T_{\text{test}}$, both containing the support set *S* and the query set *Q*. In Pretraining, a feature extractor *f_φ_*(⋅) is trained on $D_{\text{base}}$.

In meta-training phase, based on the feature extractor *f_φ_*(⋅), a meta-learner *f_θ_*(⋅) is trained on the support set of train task set $T_{\text{train}}\langle S\rangle$ by maximizing the likelihood estimation on the query set of train task set $T_{\text{train}}\langle Q\rangle$. That is,

$$\underset{\theta }{\text{max}}E_{\left(S,Q\right)\in T_{\text{train}}}\sum_{\left(x,y\right)\in Q}\text{log}\left(P\left(y|x,\omega ,S,\varphi ,\theta \right)\right)$$ (1)

where *ω* represents meta knowledge, and *φ* and *θ* represent the parameters of *f_φ_*(⋅) and *f_θ_*(⋅), respectively. We learn meta knowledge by sampling a large number of train tasks, so the optimal meta knowledge *ω* can be expressed as this:

$$\omega^{*}=\text{arg}\underset{\omega }{\text{max}}\text{log}p\left(\omega |T_{\text{train}}\right)$$ (2)

In meta-test phase, based on the optimal meta knowledge *ω*^∗^ that has been learned, the optimal meta-learner parameters *θ*^∗^ are found as following:

$$\theta^{*}=\text{arg}\underset{\theta }{\text{max}}\text{log}p\left(\theta |\omega^{*},T_{\text{test}}\right)$$ (3)

As for evaluation, we directly predict labels in $T_{\text{test}}\langle Q\rangle$ by the optimal meta-learner *f*_*θ*^∗^_(⋅) and then compare with the ground truth to evaluate the performance.

### Architecture of CharNet

**Fig. 3.**
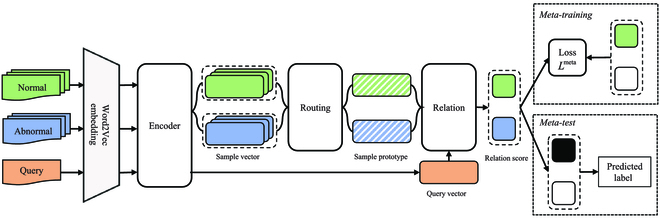
The architecture of CharNet.

Our CharNet includes 3 modules: encoder module, routing module, and relation module, which is shown in Fig. 3 (the case of 2-way 3-shot model).

#### Encoder module

In order to consider both the historical information and future information of the sequence, and let the model focus more on finding helpful information in the input data that is salient and relevant to the current output, we adopt the bidirection LSTM network with self-attention [32]. For simplicity, the encoder module receives an input sequence *x* = (*c*_1_, *c*_2_, …, *c_L_*), where *c_l_* represents the character embedding, *L* represents the length of sequence. The forward hidden state ${\vec{h}}_{t}$ and the reverse hidden state ${\overset{\leftarrow }{h}}_{t}$ are obtained by biLSTM and then concatenate ${\vec{h}}_{t}$ and ${\overset{\leftarrow }{h}}_{t}$ to obtain the hidden state *h_t_*.

$${\vec{h}}_{l}=\vec{\text{LSTM}}\left(c_{l},h_{l-1}\right)$$ (4)

$${\overset{\leftarrow }{h}}_{l}=\overset{\leftarrow }{\text{LSTM}}\left(c_{l},h_{l+1}\right)$$ (5)

$$h_{l}=\text{concatenate}\left({\vec{h}}_{l},{\overset{\leftarrow }{h}}_{l}\right)$$ (6)

We set the dimension of each LSTM unit hidden state to *u*, and the set of all hidden units state is *H* = (*h*_1_, *h*_2_, …, *h_L_*). Through a linear transformation, a variable dimension of input sequence *x* is transformed into a fixed dimension of hidden state sequence *H*. Afterwards, the self-attention mechanism is used to assign a corresponding attention score to each hidden state, which takes the set of whole hidden state *H* as input, and outputs a vector of weights *a*.

$$a=\text{softmax}\left(W_{a2}\text{tanh}\left(W_{a1}H^{T}\right)\right)$$ (7)

Here, *W*_*a*1_ ∈ *R*^*d_a_*×2*u*^ and *W*_*a*2_ ∈ *R^d_a_^* are weight matrices and *d_a_* is a hyperparameter. The output representation *e* of the encoder is the weighted sum of *a* and *H*:

$$e=\sum_{l=1}^{L}a_{l}\cdot h_{l}$$ (8)

#### Routing module

The dynamic routing algorithm is the core of this section, which is similar to the multihead attention mechanism. It can assign the weight of the samples in the support set through multiple iterations, so as to obtain a prototype vector that combines the distribution of the samples. We regard these vectors *e* obtained from the support set *S* by Eq. 8 as sample vectors *e^s^*, and the vectors e from the query set *Q* as query vectors *e^q^*. Routing module converts sample vectors $e_{\mathrm{ij}}^{s}$ to prototype vectors *p_i_* through a nonlinear mapping, where *i* = 1, …, *C* and *j* = 1, …, *K*.

Since we treat the flow as a string, and the order of the characters plays a crucial role in the model, we multiply all the sample vectors in the support set $e_{\mathrm{ij}}^{s}$ with a transformation matrix *W_s_* ∈ *R*^2*u*×2*u*^ and add a bias *b_s_*. After iteration, a most representative linear mapping can be found. Each sample prediction vector ${\hat{e}}_{\mathrm{ij}}^{s}$ is computed by:

$${\hat{e}}_{\mathrm{ij}}^{s}=\text{squash}\left(W_{s}e_{\mathrm{ij}}^{s}+b_{s}\right)$$ (9)

where *squash* is defined as Eq. 10, which not only ensures that the data is between 0-1, but also preserves the direction of the vector.

$$\text{squash}\left(x\right)=\frac{∥x{∥}^{2}}{1+∥x{∥}^{2}}\frac{x}{∥x∥}$$ (10)

In order to ensure that the prototype vector can automatically aggregate the sample feature vectors of this class in the case of very little data capacity, it is necessary to iteratively apply the dynamic routing mechanism. At each iteration, the coupling coefficients *d_i_* for each class *i* sum to 1 by softmaxing *b_i_*.

$$d_{i}=\text{softmax}\left(b_{i}\right)$$ (11)

where *b_i_* is the logits of coupling coefficients, and initialized by 0 in the first iteration. Given each sample prediction vector ${\hat{e}}_{\mathrm{ij}}^{s}$, each candidate prototype vector ${\hat{p}}_{i}$ is a weighted sum of all sample prediction vectors ${\hat{e}}_{\mathrm{ij}}^{s}$ in class *i*:

$${\hat{p}}_{i}=\sum_{j}d_{\mathrm{ij}}\cdot {\hat{e}}_{\mathrm{ij}}^{s}$$ (12)

Then the “squash” function is applied to ensure that the length of the vector output of the routing process will not exceed 1:

$$p_{i}=\text{squash}\left({\hat{p}}_{i}\right)$$ (13)

The last step of each iteration is to adjust the logarithm of the coupling coefficient *b_ij_* by means of “protocol routing”. If the modulus between the sample prediction vector ${\hat{e}}_{\mathrm{ij}}^{s}$ and the candidate prototype vector is large, that is, the two are very similar, then increase the coupling coefficient of the prediction vector, and through several iterations, a good coupling relationship can be obtained, and the final prototype vector can be obtained.

$$b_{\mathrm{ij}}=b_{\mathrm{ij}}+{\hat{e}}_{\mathrm{ij}}^{s}\cdot p_{i}$$ (14)

Formally, the dynamic routing algorithm is shown in Algorithm 2. 
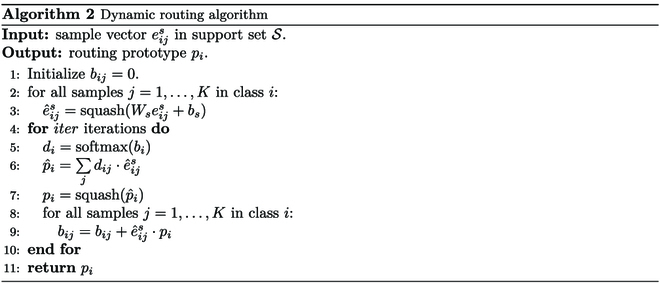


#### Relation module

We obtain the prototype vectors *p_i_* through the routing module mentioned in Routing module and use the encoder module mentioned in Encoder module to convert the samples in the query set into query vectors *e^q^*. Then, we need to measure the connection between query vectors *e^q^* and prototype vectors *p_i_*. We draw on the ideas in [21] and use neural networks to replace common mathematical distance metrics. Thus, the similarity measure between *p_i_* and *e^q^* is represented by the relation score, which is between 0 and 1.

The neural network consists of a neural tensor layer and a sigmoid layer, where the neural tensor layer outputs a relation vector as follows:

$$v\left(p_{i},e^{q}\right)=f\left(p_{i}^{T}M^{\left[1:h\right]}e^{q}\right)$$ (15)

where *M^k^* ∈ *R*^2*u*×2*u*^, *k* ∈ [1, …, *h*] is one slice of the tensor parameters and *f* is RELU activation function. The final relation score *r_iq_* between the *i*-th class and the *q*-th query is calculated by a fully connected layer activated by a sigmoid function.

$$c_{\mathrm{iq}}=\text{sigmoid}\left(W_{r}v\left(p_{i},e^{q}\right)+b_{r}\right)$$ (16)

#### Objective function

In the process of training the model, we set the mean square error loss as the loss function. The purpose is to transform a 2-way classification problem into a similarity regression problem, that is, the similarity problem between relationship scores *r_iq_* and the ground truth *y_q_*. Given the support set S with 2 classes and query set Q in an episode, the loss function is defined as:

$$L\left(S,Q\right)=\sum_{i=1}^{2}\sum_{q=1}^{n}{\left(r_{\mathrm{iq}}-1\left(y_{q}=\;=i\right)\right)}^{2}$$ (17)

All parameters of the 3 modules are trained jointly by backpropagation. The stochastic gradient descent is used on all parameters in each training episode. Our model does not need any finetuning on the classes it has never seen due to its generalization nature. The routing and comparison ability are accumulated in the model along with the training episodes.

## Results and Discussion

In this section, 2 datasets are selected to evaluate the performance of our proposed CharNet by comparing with big-data and few-shot network AD methods respectively. After that, the function of each module is analyzed through ablation study.

### Datasets

#### CICIDS2017FS

CICIDS2017 dataset [33] contains benign and the most up-to-date common attacks, which resembles the true real-world data (PCAPs). Each network traffic has been labeled by using CICFlowMeter with labeled flows based on the time stamp, source, and destination IPs, source and destination ports, protocols and attack. The dataset includes the most common attacks based on the 2016 McAfee report, such as Web based, Brute force, DoS, DDoS, Infiltration, Heart-bleed, Bot and Scant. We select 14 different types of attacks to form the data sets, 10 types of which were used as base set and 4 types as support set and query set. Then, using the task generating Algorithm 1 to generate 2-way *K*-shot tasks from each sets. The number of samples in the CICIDS2017FS is shown in Table 1.

**Table 1.** The number of samples in the CICIDS2017FS

Type of attacks	Training task		Test task	
	Support set	Query set	Support set	Query set
Botnet	2,622	1,310	-	-
DoS-GoldenEye	13,724	6,862	-	-
DoS-Hulk	308,098	154,048	-	-
DoS-slowloris	7,728	3,864	-	-
Heartbleed	16	6	-	-
Infilteration	48	24	-	-
SSH-Patator	7,864	3,930	-	-
Brute force	2,010	1,004	-	-
SQL injection	28	14	-	-
XSS	870	434	-	-
DDos	-	-	516	2,034
DoS-Slowhttptest	-	-	496	2,062
FTP-Patator	-	-	424	207
PortScan	-	-	490	2,032

#### CM2021FS

This dataset was collected from a week of real server, which contains nearly 160,000 samples in 6 different types of attacks and normal requests. The 6 classes are respectively divided into 3 (Cloud Server request, blocked IP, crawler tool) and 3 (SQL injection, Directory traversal, XSS cross-site) for pretraining tasks and meta-learning tasks. We create 2-way *K*-shot learning models on this dataset. The number of samples in the CM2021FS is shown in Table 2.

**Table 2.** The number of samples in the CM2021FS

Type of attacks	Training task		Test task	
	Support set	Query set	Support set	Query set
Cloud server access	29,796	14,896	-	-
Blocked IP	25,216	12,608	-	-
Crawler tool	16,106	8,052	-	-
SQL injection	-	-	190	810
Directory traversal	-	-	70	700
XSS cross-site	-	-	24	120

### Implementation details

#### Experiment platform

The experiments were carried out under the following hardware and software environment: Intel Core i9-10920X @3.50 GHz, 64 GB RAM, NVIDIA GeForce RTX 3090; CUDA 10.2, cuDNN 8.0, and PyTorch 1.10.2.

#### Architecture

We use Word2Vec [30] to pretrain the language coding layer on dataset $D_{\text{base}}$, where the dimension is 300 and the window size is 64. In encoder module, we set the dimension of hidden state of LSTM to 128 and the dimension of attention matrix to 64. In routing module, the iteration number *iter* is 4. In relation module, the output dimension is 100, and the activation function is Relu. After that, it is transformed into a score of [0,1] through a sigmoid layer.

#### Training details

In the pretraining stage, we pretrain the word embedding on base dataset $D_{\text{base}}$ with Word2Vec [34]. In the meta-training stage, we train the CharNet with 10,000 episodes on the support set of train task set $T_{\text{train}}\langle S\rangle$ in an episodic manner via a stochastic gradient descent with momentum of 0.9 and weight decay of 0.0005. Then, we chose the model with the highest accuracy in the query set of train task set $T_{\text{train}}\langle Q\rangle$ as the final model. In the meta-test stage, We build 2-way *K*-shot (*K* = [5, 10]) models on 2 datasets to simulate the scenario of the network AD.

### Discussion of results

In this section, we provide an overview of several most recent deep learning and machine learning algorithms in network AD and discuss the detection results and the number of samples. Then, we compare the proposed routing network with other few-shot learning methods. After that, the ablation study is conducted to evaluate the effect of each module.

#### Comparison with big-data methods

In previous studies, many scholars have achieved excellent performance using machine learning and deep learning algorithms based on large amounts of data. To ensure the fairness of the experiments, we made a comparison between CharNet with other existing researches that used the same public benchmark dataset CICIDS2017. At the same time, we also conduct few-shot experiments on the private dataset CM2020FS, the precision and recall were 94.29% and 96.56% for *K* = 5, and 98.56% and 97.68% for *K* = 10. The results are shown in Table 3.

**Table 3.** Comparison of detection result and the number of samples in the proposed method and big-data methods

Approach	Method	Dataset	Type	Number of samples	Acc (%)	Recall (%)
Machine learning	GA-based Adaptive Method (2018) [40]	CICIDS2017	GA + clustering	760,056	N/A	92.85
	Multilayer ensemble SVM (2018) [5]	CICIDS2017	SVM	N/A	N/A	94.94
	DT and Rule Based IDS (2019) [37]	CICIDS2017	REP Tree + random forest	40,000	96.66	94.47
	GBT-based Big Data Method (2019) [36]	CICIDS2017	Gradient Boosted Tree	1,000,000	99.97	N/A
	Improved AdaBoost-based IDS (2019) [6]	CICIDS2017	AdaBoost	158,021	81.83	100.0
	Multi-Stage Optimized ML-based IDS (2021) [4]	CICIDS2017	KNN + random forest	2,830,540	99.99	99.00
Deep learning	Flow-based deep learning method (2018) [14]	CICIDS2017	CNN + LSTM	1,028,007	98.87	98.83
	SU-IDS(2018) [38]	CICIDS2017	Autoencoder	40,000	71.02	N/A
	Deep Hierarchical IDS (2019) [41]	CICIDS2017	CNN + LSTM	553,850	99.91	99.92
	Random attention capsule (2020) [42]	CICIDS2017	Attention + capsule	863,240	98.60	98.61
	DNN-kNN IDS (2020) [34]	CICIDS2017	DNN + kNN	225,745	99.63	99.69
	CLAIRE (2021) [11]	CICIDS2017	Autoencoder + CNN	100,000	98.01	95.20
Ours	CharNet	CICIDS-2017FS	Siamese + Routing	5	95.94	98.78
	CharNet	CICIDS-2017FS	Siamese + Routing	10	99.87	99.98
	CharNet	CM2021FS	Siamese + Routing	5	94.29	96.56
	CharNet	CM2021FS	Siamese + Routing	10	98.56	97.68

The noteworthy observation is that the overwhelming majority of researches, whether machine learning or deep learning, are based on “big data”. As can be seen from Table 3, the accuracy and recall of these researches are almost above 95%, for example, Multi-Stage Optimized ML-based AD (2021) [4] even achieves 99.99% accuracy and 99.00% recall. However, their sample size has reached hundreds of thousands, or even millions, which requires tremendous human efforts to collect, process and label these data manually. In addition, it is difficult for us to quickly identify new and ever-changing attacks.

As shown in Table 3, CharNet (*K* = 5) only outperforms GA-based Adaptive Method [35], Multilayer ensemble SVM [5], and SU-AD [36] in both accuracy and recall, while CharNet (*K* = 10) outperforms all methods except GBT-based Big Data Method [37], Improved AdaBoost-based AD [6] and Deep Hierarchical AD [38]. With a slight decline about 4% and 0.8% in accuracy and recall (*K* = 5), and about 0.1% and 0.02% in accuracy and recall (*K* = 10), required training samples of CharNet is much fewer than these method. It should be noted that the result obtained by CharNet is on the basis of only 5 and 10 labeled samples used in training process, which drastically reduces the cost of data collection and manual labeling.

#### Comparison with few-shot learning methods

We compare CharNet with some state-of-the-art metric-based approaches of network AD, such as FC-Net, DF-Net, GP-Net, and FS-AD. FC-Net [18] is based on RelationNet to implement few-shot traffic classification and achieves good performance in its experimental setting. DF-Net [23] takes use of siamese capsule network for AD with imbalanced traning data. A relative position mechanism and a global-enhanced feature extractor are designed in GP-Net [26] to capture the relationship between arbitrary 2-byte payload sequences. FS-AD [27] adopts autoencoder, CNN, and euclidean distance metric module in series in the few-shot frame.

To ensure fairness, we compare the performance of our method and the baseline methods on the same benchmark dataset CICIDS2017FS, the result is shown as Table 4. In order to be consistent with the setting of FC-Net, we did 2 experiments with *K* = 5 and *K* = 10. It can be found that our proposed network achieves the highest precision and recall in both *k* = 5 and *k* = 10 experiments. In the 2-way 5-shot experiment, the accuracy of CharNet exceeds the state-of-the-art methods by 1.3%∼2.34%, and the recall rate exceeds 0.18%∼4.55%. In the 2-way 10-shot experiment, the accuracy exceeds the advanced method by 2.36%∼4.48%, and the recall rate exceeds 0.81%∼3.58%. It is worth noting that our method beats FC-Net, which demonstrates that the routing-based prototype is more effective than the mean-based prototype.

**Table 4.** Few-shot experimental result on CICIDS2017FS

Methods	Two-way 5-shot		Two-way 10-shot	
	Acc (%)	Recall (%)	Acc (%)	Recall (%)
FC- Net [18]	94.13	98.49	95.39	98.19
DF- Net [23]	93.87	96.29	95.56	96.4
GP- Net [26]	94.58	94.23	96.40	96.96
FS- AD [27]	93.60	98.60	97.51	99.17
CharNet	95.94	98.78	99.87	99.98

### Ablation study

The experiments are conducted on CICIDS2017FS and CM2021FS to evaluate the effect of each module, i.e., encoder module, routing module, relation module, and pretraining. Specifically, (a) we change the backbone network of the encoder to explore the best feature extractor; (b) for the routing module, we change the number of iterations *iter* of the routing algorithm to find the best routing-based prototype in different datasets and also compare with the mean-based prototype. (c) We replace the relation module with cosine distance metric and euclidean distance metric.

#### The effect of encoder module

In the experiment of the encoder module, the different backbone network are applied, such as biLSTM, LSTM, CNN, and Tranformer. The results are shown in Table 5. It can be found that (a) the accuracy of biLSTM with attention on both datasets outperform the other 3 backbone networks, while the transformer is not too far apart, whose recall achieves the best results on CICIDS2017FS; (b) the attention mechanism plays a role and helps biLSTM improve the accuracy and recall by around 1%; (c) LSTM with attention is around 4% in precision and 3% in recall lower than biLSTM with attention, which means that the informal text such as network traffic requires bidirectional semantics to better express the information in it.

**Table 5.** Ablation study with encoder module on 2-way 10-shot tasks

Encoder module	CICIDS2017FS		CM2021FS	
	Acc (%)	Recall (%)	Acc (%)	Recall (%)
biLSTM + attention	99.87	99.85	98.56	98.92
LSTM + attention	95.60	96.07	94.62	95.16
CNN + attention	97.83	97.98	97.58	97.98
Transformer	98.67	99.87	98.47	98.86
biLSTM	98.60	98.76	98.19	97.29

**Fig. 4.**
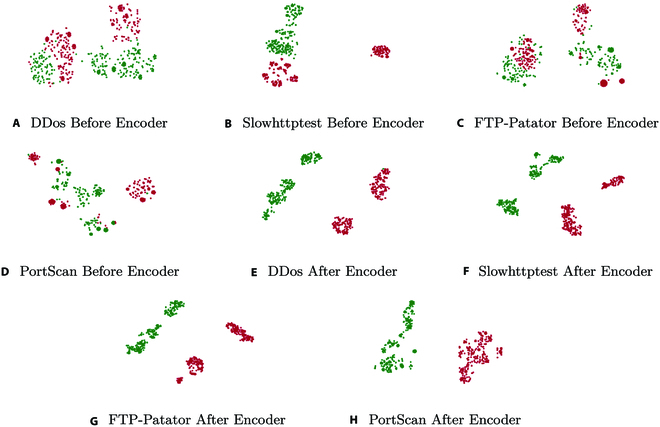
Effect of Encoder under CICIDS2017FS (the green dots indicate normal traffic, and the red dots indicate abnormal traffic). (A) DDos before Encoder. (B) Slowhttptest before Encoder. (C) FTP-Patator before Encoder. (D) PortScan before Encoder. (E) DDos after Encoder. (F) Slowhttptest after Encoder. (G) FTP-Patator after Encoder. (H) PortScan after Encoder.

In Fig. 4, it shows the t-stochastic neighbor embedding [39] visualization before and after encoder module. We carry out the test task of CICIDS2017FS, which includes 4 categories: DDos, Slowhttptest, FTP-Patator, and PortScan. We can see that the vectors after encoder are more separable, demonstrating the effectiveness of encoder to separate the solution space.

#### The effect of routing module

To explore the effect of iterations *iter* on the routing module, we set *iter* from 1 to 6 on CICIDS2017FS and CM2021FS. In addition, the routing module is removed and replaced the mean-based prototype. According to the result in Table 6, we observe that (a) the best performance is achieved when we used 4 and 5 iterations, and more rounds of iterations did not further improve the performance; (b) the best performance of routing module exceeds the mean-based prototype by around 1%, which indicates that the routing module shows effectiveness.

**Table 6.** Ablation study with routing module on 2-way 10-shot tasks

Routing module	iter	CICIDS2017FS		CM2021FS	
		Acc (%)	Recall (%)	Acc (%)	Recall (%)
Routing	1	97.41	98.78	93.94	97.56
Routing	2	97.52	98.89	95.78	98.75
Routing	3	98.52	98.64	97.62	98.24
Routing	4	99.87	99.85	98.34	98.92
Routing	5	98.97	99.37	98.56	99.13
Routing	6	98.02	98.67	97.92	98.36
Mean	-	98.89	99.12	96.98	97.38

#### The effect of relation module

For the relation module, we draw on the idea of relation network [21] and use neural network training to obtain a learnable nonlinear similarity measure function, thereby constructing an end-to-end network structure. The experimental results of the relaton module are shown in Table 7, from which we find that the relation module outperform the cosine and euclidean distance metric on both datasets.

**Table 7.** Ablation study with relation module on 2-way 10-shot tasks

Relation module	CICIDS2017FS		CM2021FS	
	Acc (%)	Recall (%)	Acc (%)	Recall (%)
Relation	99.87	99.85	98.56	98.92
Cosine	96.46	98.69	97.98	98.88
Euclidean	97.04	99.24	97.52	98.58

#### The effect of pretraining

**Fig. 5.**
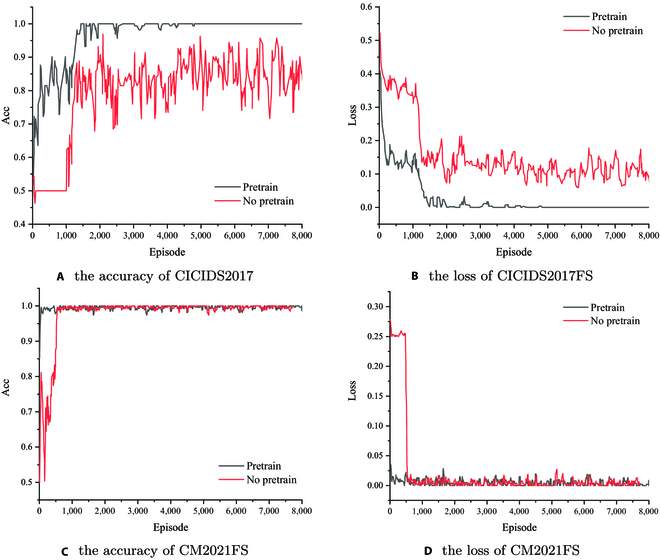
Effect of pretraining under CICIDS2017FS and CM2021FS. (A) The accuracy of CICIDS2017. (B) The loss of CICIDS2017F. (C) The accuracy of CM2021FS. (D) The loss of CM2021FS.

To pursue faster training speed, we consider adding pretraining before the encoder module. Figure 5 shows the accuracy and loss curves with and without pretraining in CICIDS2017FS and CM2021FS, where *iter* is set to 4 and 5 respectively, and *episode* is set to 8,000. It can be seen intuitively that the convergence can be faster with pretraining, indicating that the pretraining can effectively extract the text information in the traffic log.

## Conclusion

In this paper, we propose the CharNet, a novel neural model for few-shot network AD prototype. For this purpose, a basic binary classification task was defined and a pair of network traffic samples including a normal unaffected sample and a malicious one were constructed for learning. The routing module combines the dynamic routing algorithm with a meta-learning prototype, and the routing mechanism finds a more accurate prototype through multiple iterations, making our model more general to recognize unseen classes. The experiment results show that the proposed model outperforms the existing state-of-the-art few-shot network AD models, and only need 5 or 10 samples to get a high-accuracy model. We found that both the pretraining and encoder contribute tremendously to the few-shot learning tasks.

## Data Availability

The open dataset CICIDS2017FS can be accessed through the following link: https://www.unb.ca/cic/datasets/ids-2017.html.
